# [^89^Zr]Zr-PSMA-617 PET/CT characterization of indeterminate [^68^Ga]Ga-PSMA-11 PET/CT findings in patients with biochemical recurrence of prostate cancer: lesion-based analysis

**DOI:** 10.1186/s40644-024-00671-1

**Published:** 2024-02-22

**Authors:** Florian Rosar, Caroline Burgard, Elena Larsen, Fadi Khreish, Robert J. Marlowe, Andrea Schaefer-Schuler, Stephan Maus, Sven Petto, Mark Bartholomä, Samer Ezziddin

**Affiliations:** 1 Department of Nuclear Medicine, Saarland University– Medical Center, Kirrberger Str. 100, Geb. 50, D-66421 Homburg, Germany; 2 Spencer-Fontayne Corporation, Jersey City, NJ USA

**Keywords:** Prostate cancer, Biochemical recurrence, Positron emission tomography/computed tomography (PET/CT), Indeterminate findings, Prostate-specific membrane antigen (PSMA), Zirconium-89 (^89^Zr)

## Abstract

**Background:**

The state-of-the-art method for imaging men with biochemical recurrence of prostate cancer (BCR) is prostate-specific membrane antigen (PSMA)-targeted positron emission tomography/computed tomography (PET/CT) with tracers containing short-lived radionuclides, e.g., gallium-68 (^68^Ga; half-life: ∼67.7 min). However, such imaging not infrequently yields indeterminate findings, which remain challenging to characterize. PSMA-targeted tracers labeled with zirconium-89 (^89^Zr; half-life: ∼78.41 h) permit later scanning, which may help in classifying the level of suspiciousness for prostate cancer of lesions previously indeterminate on conventional PSMA-targeted PET/CT.

**Methods:**

To assess the ability of [^89^Zr]Zr-PSMA-617 PET/CT to characterize such lesions, we retrospectively analyzed altogether 20 lesions that were indeterminate on prior [^68^Ga]Ga-PSMA-11 PET/CT, in 15 men with BCR (median prostate-specific antigen: 0.70 ng/mL). The primary endpoint was the lesions’ classifications, and secondary endpoints included [^89^Zr]Zr-PSMA-617 uptake (maximum standardized uptake value [SUV_max_]), and lesion-to-background ratio (tumor-to-liver ratio of the SUV_max_ [TLR]). [^89^Zr]Zr-PSMA-617 scans were performed 1 h, 24 h, and 48 h post-injection of 123 ± 19 MBq of radiotracer, 35 ± 35 d post-[^68^Ga]Ga-PSMA-11 PET/CT.

**Results:**

Altogether, 6/20 previously-indeterminate lesions (30%) were classified as suspicious (positive) for prostate cancer, 14/20 (70%), as non-suspicious (negative). In these two categories, [^89^Zr]Zr-PSMA-617 uptake and lesional contrast showed distinctly different patterns. In positive lesions, SUV_max_ and TLR markedly rose from 1 to 48 h, with SUV_max_ essentially plateauing at high levels, and TLR further steeply increasing, from 24 to 48 h. In negative lesions, uptake, when present, was very low, and decreasing, while contrast was minimal, from 1 to 48 h. No adverse events or clinically-relevant vital signs changes related to [^89^Zr]Zr-PSMA-617 PET/CT were noted during or ~ 4 weeks after the procedure.

**Conclusions:**

In men with BCR, [^89^Zr]Zr-PSMA-617 PET/CT may help characterize as suspicious or non-suspicious for prostate cancer lesions that were previously indeterminate on [^68^Ga]Ga-PSMA-11 PET/CT.

**Trial registration:**

Not applicable.

## Background

The state-of-the-art method for imaging men with biochemical recurrence of prostate cancer (BCR) is prostate-specific membrane antigen (PSMA)-targeted positron emission tomography/computed tomography (PET/CT) [1–3]. Most widely used for this purpose at present are PSMA-targeted tracers containing short-lived radionuclides, e.g., gallium-68 (^68^Ga; half-life: ∼67.7 min) or fluorine-18 (^18^F, half-life ∼109.8 min) [4–8].

Besides improving sensitivity and specificity of prostate cancer imaging, PSMA-targeted PET/CT has reduced the rate of equivocal findings compared to that seen with the previous standard procedures, CT and bone scan. For example, in the proPSMA study (*N* = 295) in the primary staging setting, [^68^Ga]Ga-PSMA-11 PET/CT was associated with a 7% (95% confidence interval: 4–13%) rate of equivocal scans, versus 23% (95% confidence interval: 17–31%) for the older modalities [9]. Nonetheless, indeterminate lesions remain an appreciably frequent challenge in whole-body prostate cancer staging. In many cases, characterization of such lesions may be decisive in optimizing treatment planning, and is therefore of high clinical importance [10].

PSMA-targeted PET/CT with tracers incorporating zirconium-89 (^89^Zr) are a potential means of characterizing lesions that are indeterminate on conventional PSMA-targeted PET/CT, i.e., that conducted using ^68^Ga-labeled or ^18^F-labeled radiotracers. Due to the much longer half-life of ^89^Zr, ∼ 78.41 h, tracers conjugated with that radionuclide allow much later imaging, i.e., at ≥ 24 h post-injection. For this reason, ^89^Zr tracers may clearly visualize lesions that more slowly internalize PSMA ligands, e.g., those with weak PSMA expression or poor perfusion [11–16]. Additionally, late scanning allows greater time for radiopharmaceutical clearance from non-target tissue, thereby increasing tumor-to-background ratio [14].

We, our collaborators from Radboud University Medical Center/the University of Nijmegen, and others have reported that PET/CT at ≥ 24 h post-injection with [^89^Zr]Zr-PSMA-617 or other ^89^Zr-labeled tracers frequently detects lesions suspicious for prostate cancer that were not apparent on conventionally-acquired PET/CT images [11–16]. Our group also reported promising preliminary experience using [^89^Zr]Zr-PSMA-617 in 3 patients with indeterminate findings on [^68^Ga]Ga-PSMA-11 PET/CT [13]. However, to our knowledge, the literature contains no additional data regarding the ability of [^89^Zr]Zr-PSMA-617 PET/CT to characterize as suspicious or non-suspicious for prostate cancer lesions that were indeterminate on conventional PSMA-targeted PET/CT. We therefore sought to assess the use of this novel imaging modality in a larger cohort of patients with indeterminate findings on PET using tracers with short-lived radionuclides.

## Methods

### Study design and endpoints

This was a retrospective, lesion-based analysis. The primary endpoint was the visual classification on [^89^Zr]Zr-PSMA-617 PET/CT of lesions that on a prior [^68^Ga]Ga-PSMA-11 PET/CT scan, had been judged to be indeterminate. Additional secondary endpoints comprised the values of and changes over time in [^89^Zr]Zr-PSMA-617 PET variables for each previously-indeterminate lesion, and the number and sites of lesions detected on [^89^Zr]Zr-PSMA-617 PET/CT, but not on [^68^Ga]Ga-PSMA-11 PET/CT.

The remaining secondary endpoints were near-term safety, i.e., side effects or vital signs abnormalities observed during or shortly after the procedure that we deemed to be related to [^89^Zr]Zr-PSMA-617 PET/CT, and results of follow-up of patients in the study sample.

### Patients and ethics

The cohort included 15 consecutive men with BCR who had ≥ 1 indeterminate lesion on prior [^68^Ga]Ga-PSMA-11 PET/CT. BCR was defined as increasing prostate-specific antigen (PSA) after primary (curative-intent) treatment. Imaging took place between 25 October 2021 and 6 February 2023 at Saarland University Medical Center, Homburg, Germany. The [^68^Ga]Ga-PSMA-11 PET/CT was conducted following standard procedures [17], ∼ 1 h after infusion of, on average, 151 ± 25 MBq of radiotracer. Indeterminate lesions were defined as those that could not be clearly attributed to pathological or physiological uptake, e.g., visually faint foci at typical sites of prostate cancer recurrence, foci at sites unusual for recurrence, or foci that lacked an apparent anatomical correlate on the concurrent CT. [^68^Ga]Ga-PSMA-11 PET/CT images were classified visually by consensus among three experienced nuclear medicine specialists (SE, FK, FR). To eliminate a potential confounder in [^89^Zr]Zr-PSMA-617 scan interpretation, patients were excluded from the analysis if their prostate cancer treatment changed in the time between the [^68^Ga]Ga-PSMA-11 scan and the [^89^Zr]Zr-PSMA-617 scan.

Table 1 summarizes patient and imaging characteristics of the study sample. This cohort was typically middle-aged to elderly, with Gleason stage 8 or 9 disease in 60% of cases. PSA values were (median [minimum–maximum] 0.70 [0.10–10.2] ng/mL). Data regarding 3/15 patients were previously published [13].

**Table 1:** Patient and imaging characteristics of 15 men with indeterminate [^68^Ga]Ga-PSMA-11 PET/CT

Characteristic	Value
Age [yr]	
Median (min.–max.)	71 (59–77)
PSA [ng/mL]	
Median (min.–max.)	0.70 (0.10–10.2)
PSA doubling time category, % (n)	
<3 mo.	33% (5)
3–6 mo.	33% (5)
7–12 mo.	13% (2)
>12 mo.	20% (3)
Gleason Score category, % (n)	
6	7% (1)
7a	13% (2)
7b	20% (3)
8	27% (4)
9	33% (5)
Primary treatment, % (n)	
Prostatectomy alone	40% (6)
Prostatectomy + lymphadenectomy	47% (7)
Radiation therapy	13% (2)
Additional treatments before study imaging, % (n)	
Radiation therapy	20% (3)
ADT	20% (3)
Number of indeterminate lesions on [^68^Ga]Ga-PSMA-11 PET/CT	
Total	20
Median (min.–max.) per patient	1 (1–3)
Percentage (number) of patients with multiple indeterminate lesions	27% (4)
Sites(s) of indeterminate lesions on [^68^Ga]Ga-PSMA-11 PET/CT, % (n)	
Local	20% (4)
Lymph node	40% (8)
Bone	40% (8)

Because of rounding, percentages may not add up to 100% for certain characteristics

ADT: androgen deprivation therapy or antiandrogen therapy; max.: maximum; min.: minimum; PSA: prostate-specific antigen; SD: standard deviation

Altogether 20 indeterminate lesions had been described on [^68^Ga]Ga-PSMA-11 PET/CT, 4 located in the prostate bed, 8 in or adjacent to lymph nodes, and 8 in the skeleton. Each patient had a limited number of such lesions: 1 each in 11 men, 2 each in 3 patients, and 3 in 1 patient. Besides the indeterminate lesion(s), 7/15 (47%) men had lesions that could be clearly classified as suspicious on [^68^Ga]Ga-PSMA-11 PET/CT; in 8/15 (53%) patients, the indeterminate lesion(s) were the only findings on the conventional PSMA-targeted scan.

[^89^Zr]Zr-PSMA-617 PET/CT was performed on a compassionate use basis under the German Pharmaceutical Act § 13 (2b). Attending nuclear medicine physicians had direct responsibility for the procedure, including ordering the radiopharmaceutical. The analysis conformed to the Declaration of Helsinki and received approval from the Institutional Review Board of the Ärztekammer des Saarlandes/Saarbrücken (approval number: 170/22, approval date: 13 September 2022). Written consent for [^89^Zr]Zr-PSMA-617 PET/CT was obtained from all patients after they received detailed information on the risks of radiation exposure associated with this procedure, and on the potential for side effects of the novel radiotracer. The consent also permitted the patient’s data to be reported in de-identified form in scientific publications.

### [^89^Zr]Zr-PSMA-617 PET/CT

[^89^Zr]Zr-PSMA-617 PET/CT took place 35 ± 35 (median [minimum–maximum] 25 [7–140]) d after the [^68^Ga]Ga-PSMA-11 PET/CT. One hr, 24 h, and 48 h after intravenous injection of [^89^Zr]Zr-PSMA-617, whole-body PET/CT images, extending from vertex to mid-femur, were acquired. The mean ± standard deviation (SD) [^89^Zr]Zr-PSMA-617 activity was 123 ± 19 MBq, the median (minimum–maximum) activity was 125 (85–157) MBq, and radiotracer administration was immediately followed by a 500-mL NaCl 0.9% infusion. Patients were asked to void before each image acquisition. [^89^Zr]Zr-PSMA-617 was manufactured in-house [13]. Imaging was performed on a Biograph mCT 40 system (Siemens Medical Solutions, Knoxville, TN, USA). Acquisition time was 3 min/bed position for the 1-hr scan, 4 min/bed position for the 24-hr scan, and 5 min/bed position for the 48-hr scan. For attenuation correction and anatomical localization, low-dose CT was carried out using a 120-keV x-ray tube voltage and tube current modulation with CARE Dose4D software (Siemens Healthineers, Erlangen, Germany), with 30 mAs as the reference. A soft tissue kernel (B31f/Be32) and a slice thickness of 5 mm (increment: 2–4 mm) were employed for data reconstruction. PET emission data also underwent decay correction, random correction, and scatter correction. An iterative 3-dimensional ordered-subset expectation maximization algorithm (3 iterations; 24 subsets) with Gaussian filtering to a transaxial resolution of 5 mm at full width at half maximum was applied to reconstruct the PET images. Matrix and pixel sizes were 200 × 200 and 3.0 mm, respectively.

### [^89^Zr]Zr-PSMA-617 PET/CT image interpretation

[^89^Zr]Zr-PSMA-617 PET/CT findings were classified visually, by consensus among the same nuclear medicine specialists who had interpreted the [^68^Ga]Ga-PSMA-11 PET/CT images. Lesions were considered to be positive on [^89^Zr]Zr-PSMA-617 PET/CT if they were visible on the 24-h scan and/or the 48-h scan as foci of clear uptake. Lesions were deemed to be negative if they could not be visualized on late imaging. Because [^89^Zr]Zr-PSMA-617 PET/CT images were interpreted within everyday practice and not a clinical study, the readers could access the patient’s prostate cancer-related and other medical history and prior images.

### Calculation of PET variables

In separate analyses of lesions that respectively had been classified as indeterminate or as positive on the prior conventional scan, two key on [^89^Zr]Zr-PSMA-617 PET variables were measured. First, the maximum standardized uptake value (SUV_max_), reflecting lesional uptake of [^89^Zr]Zr-PSMA-617, was determined. SyngoVia Enterprise VB 60 software (Siemens) was used. Second, the tumor-to-liver ratio (TLR), reflecting lesional contrast, was calculated. TLR was defined as the SUV_max_ of the lesion divided by the mean standardized uptake value (SUV_mean_) of the tissue representing background, in this analysis, healthy liver. The SUV_mean_ was calculated in a spherical volume of interest within the liver.

### Monitoring for potential adverse events related to [^89^Zr]Zr-PSMA-617 PET/CT

Adverse events and clinically-relevant vital signs abnormalities that were believed to be associated with [^89^Zr]Zr-PSMA-617 PET/CT and were noted by health care professionals, the patient, or both during imaging and up to 4 weeks thereafter were recorded. In telephone calls made shortly after scanning and/or after the first follow-up visit, patients were questioned about specific side effects and, in open-ended fashion, about the occurrence of side effects in general.

### Patient follow-up

Data were compiled regarding subsequent therapy and biochemical follow-up of patients in the study sample. This compilation was performed via retrospective analysis of medical records or via personal interview.

### Statistics

Data are presented as descriptive statistics including, as appropriate, mean ± SD, median (minimum–maximum), and number (percentage) or vice versa.

## Results

**Fig. 1:**
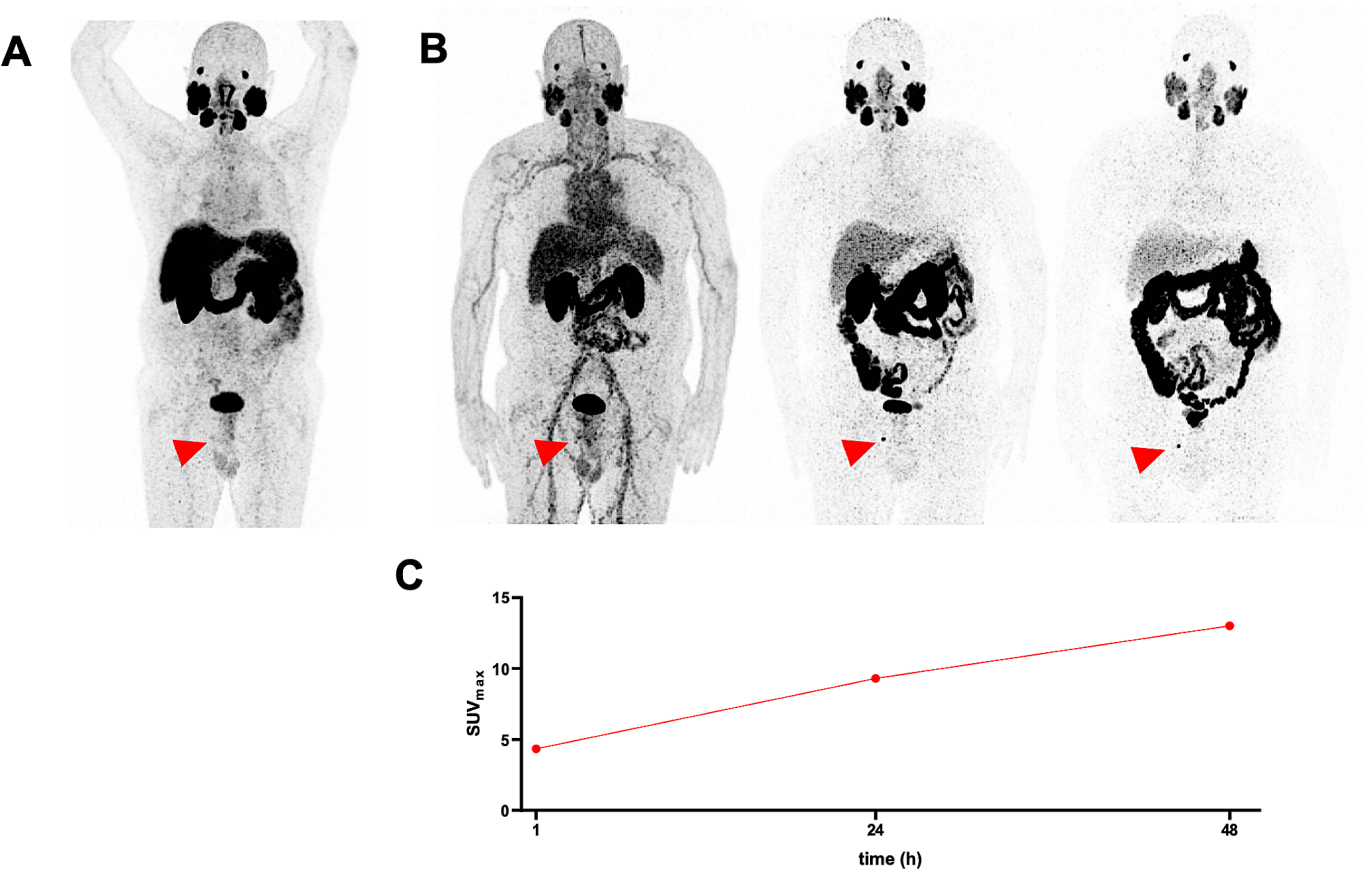
Maximum intensity projection (MIP) images of a patient with biochemical recurrence of prostate cancer on A) [68Ga]Ga-PSMA-11 PET/CT 1 h post-injection and B) (right to left) [89Zr]Zr-PSMA-617 PET/CT 1 h, 24 h, and 48 h post-injection. As denoted by the red arrows, a lesion faintly visible on the [68Ga]Ga-PSMA-11 scan, although not clearly discernible on the 1-hr [89Zr]Zr-PSMA-617 image, was clearly discernible as a presumed bone metastasis on the 24-hr and 48-hr [89Zr]Zr-PSMA-617 scans. Supporting the visual findings, [89Zr]Zr-PSMA-617 uptake, reflected by C) the SUVmax curve for the lesion, showed a sharp increase from 1 to 24 h, and then a slower increase from 24 to 48 h

Of altogether 20 lesions considered to be indeterminate for prostate cancer on [^68^Ga]Ga-PSMA-11 PET/CT, 6 (30%) were classified as suspicious (positive) and the remaining 14 (70%) as non-suspicious (negative) on [^89^Zr]Zr-PSMA-617 PET/CT. Figure 1 shows [^89^Zr]Zr-PSMA-617 PET/CT images at 1 h, 24 h, and 48 h post-injection, and the corresponding [^68^Ga]Ga-PSMA-11 PET/CT scan 1 h post-injection from a patient whose indeterminate lesion was confirmed to be suspicious (positive) on [^89^Zr]Zr-PSMA-617 PET/CT; also illustrated is a quantitative analysis of this lesion.

As seen in Table 2, the lesions classified as positive on the [^89^Zr]Zr-PSMA-617 scan comprised 3 of 4 possible local recurrences, 1 of 8 possible lymph node metastases, and 2 of 8 possible bone metastases.

**Table 2:** Classification of [^68^Ga]Ga-PSMA-11 PET/CT-indeterminate lesions and new positive findings on [^89^Zr]Zr-PSMA-617 PET/CT

Lesion type	Number of indeterminate lesions on [^68^Ga]Ga-PSMA-11 PET/CT	Classification of [^68^Ga]Ga-PSMA-11 PET/CT-indeterminate lesions on [^89^Zr]Zr-PSMA-617 PET/CT		New positive findings on [^89^Zr]Zr-PSMA-617 PET/CT
		Positive, n (% of category)	Negative, n (% of category)	
Any	20	6/20 (30%)	14/20 (70%)	11
Local	4	3/4 (75%)	1/4 (25%)	3
Lymph node	8	1/8 (13%)	7/8 (88%)	8
Bone	8	2/8 (25%)	6/8 (75%)	0

Due to rounding, percentages may not add up to 100% for certain categories of lesions

**Fig. 2:**
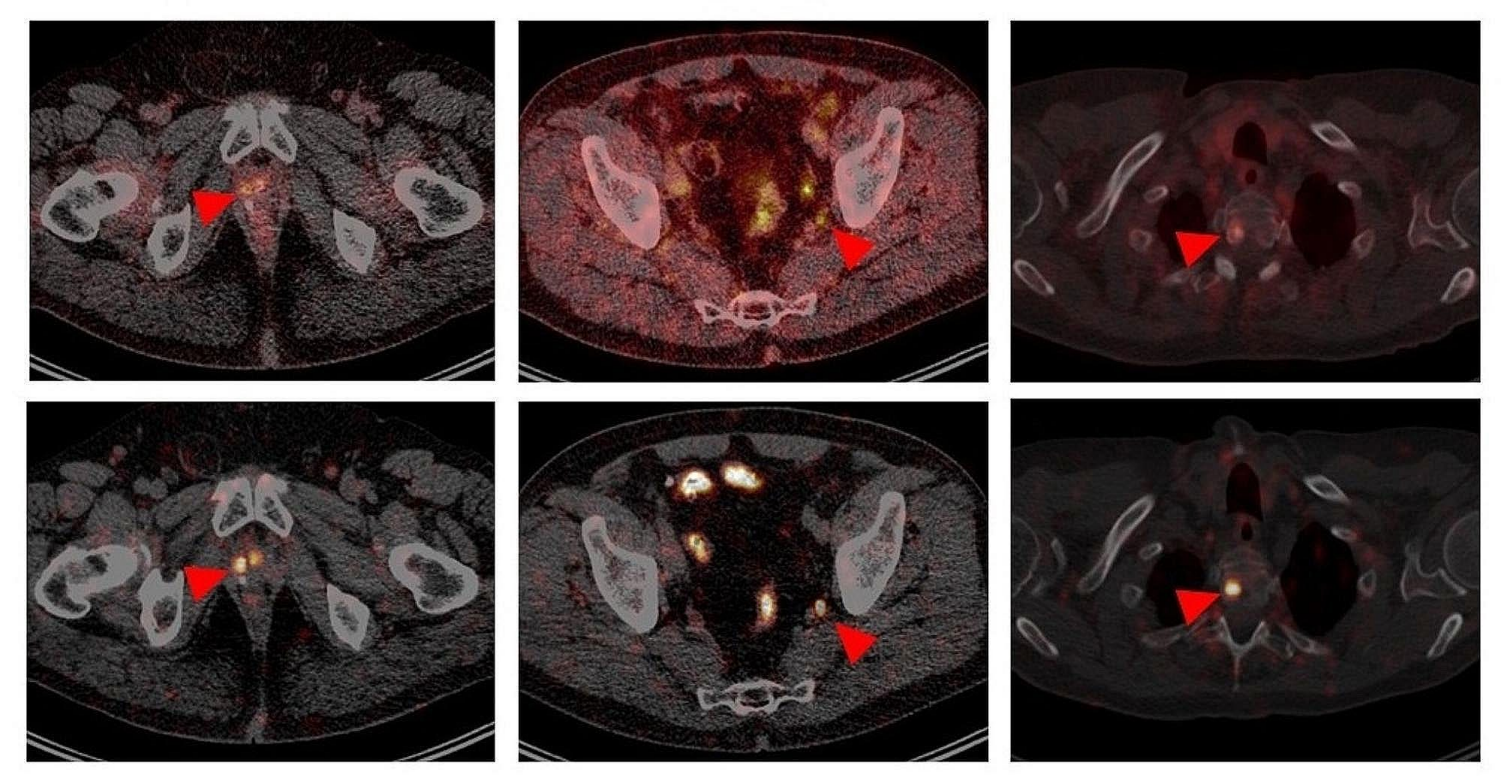
Representative transversal slice images, from 3 different patients (one per column), showing lesions (indicated by red arrows) that were indeterminate on [^68^Ga]Ga-PSMA-11 PET/CT (top row) but could be visually classified as suspicious (positive) for, respectively (bottom row, left to right), local recurrence, lymph node metastasis, and bone metastasis of prostate cancer on [^89^Zr]Zr-PSMA-617 PET/CT (48-hr scans shown here)

**Fig. 3:**
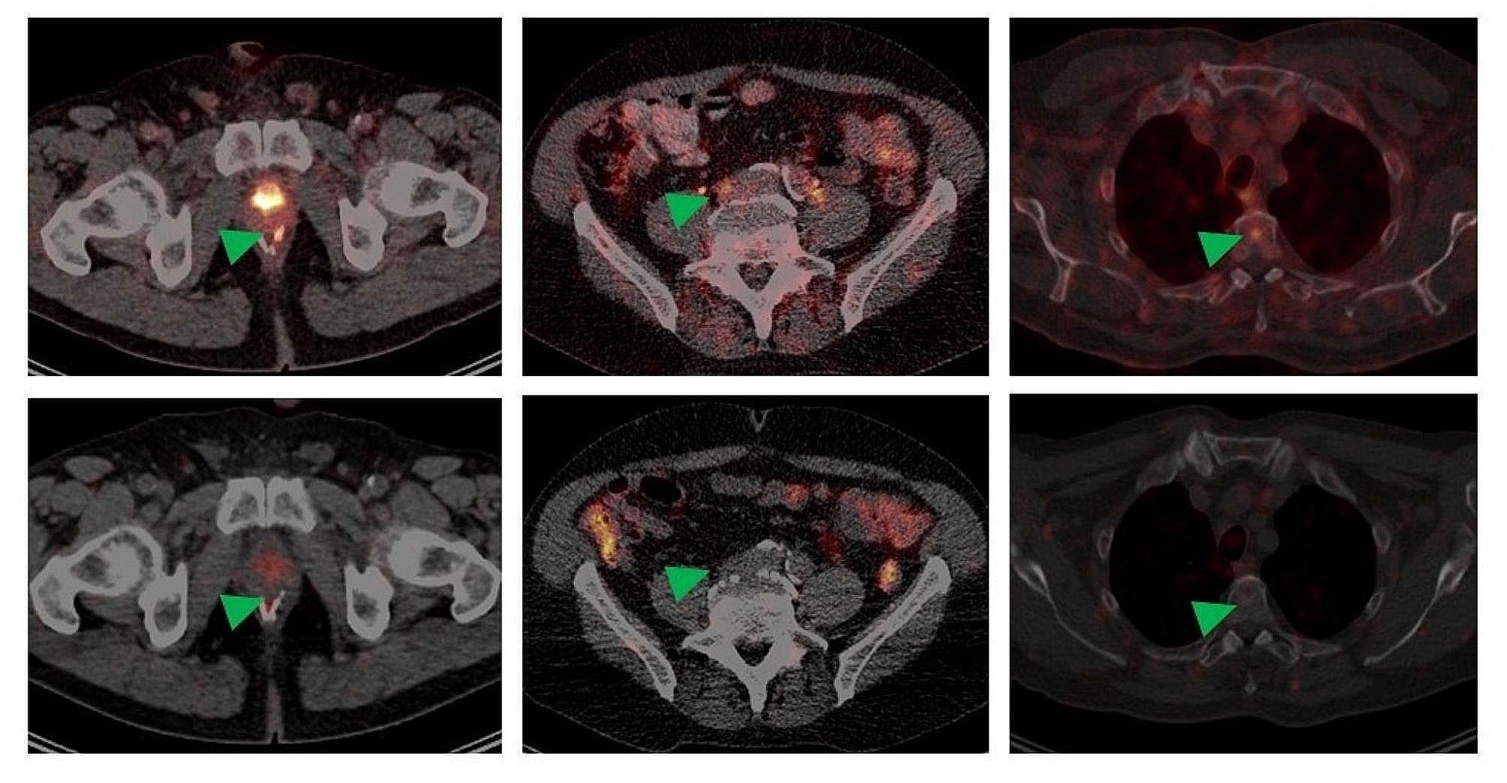
Representative transversal slice images, from 3 different patients (one per column) showing lesions (indicated by green arrows), that were indeterminate on [^68^Ga]Ga-PSMA-11 PET/CT (top row) but could be visually classified as non-suspicious (negative) for prostate cancer on [^89^Zr]Zr-PSMA-617 images (48-hr scan shown here). The lesions indeterminate on [^68^Ga]Ga-PSMA-11 PET/CT were considered possibly suspicious for local recurrence, lymph node metastasis, and bone marrow metastasis, respectively (left to right), of prostate cancer

Representative images of positive and negative lesions for each category of anatomical site appear in Figs. 2 and 3.

**Fig. 4:**
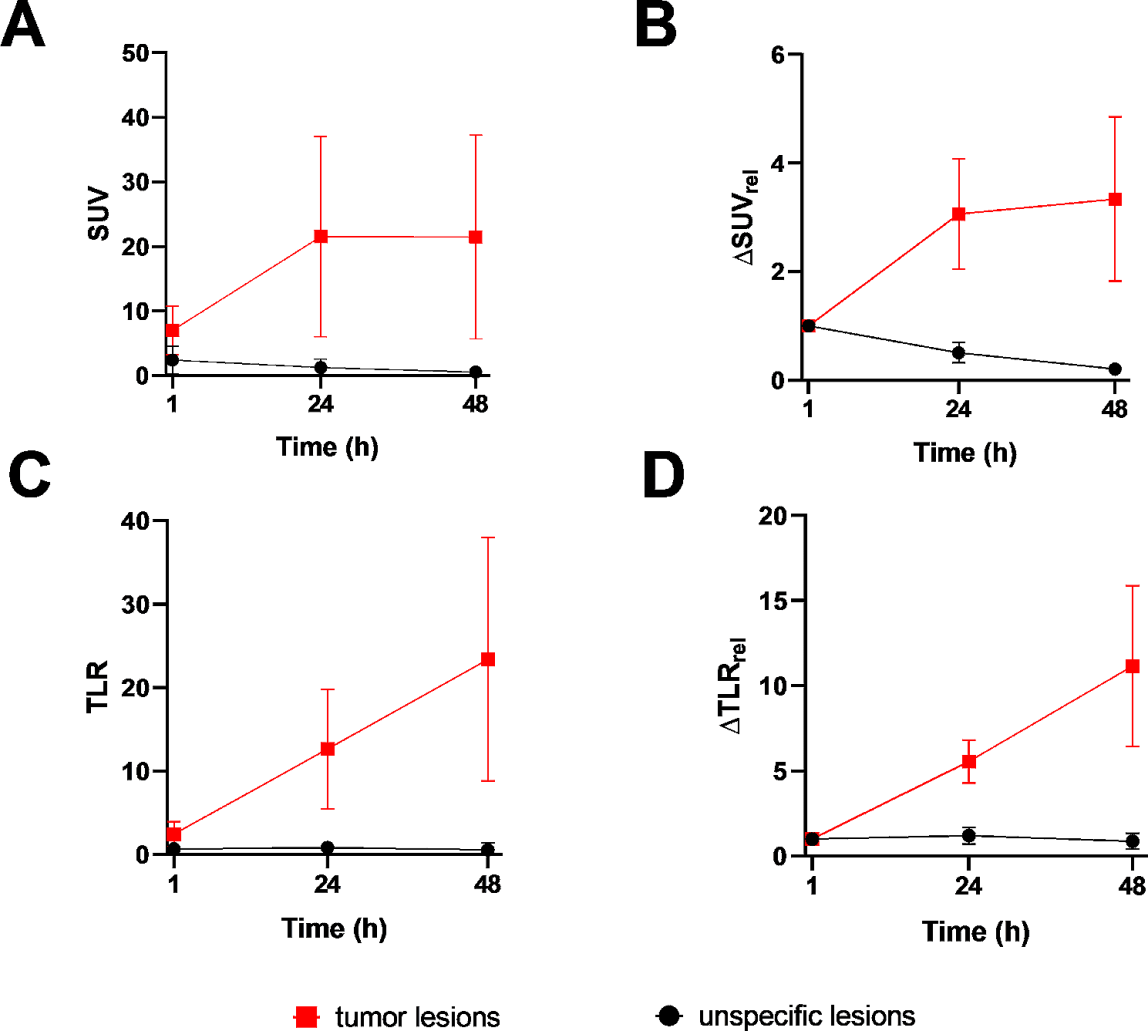
[^89^Zr]Zr-PSMA-617 PET variables by scan time and their relative changes over time of [^68^Ga]Ga-PSMA-11-indeterminate lesions visually classified as positive (*n* = 6 lesions) versus negative (*n* = 14 lesions) on [^89^Zr]Zr-PSMA-617 PET/CT. (**A**) SUV_max_, (**B**) ∆SUV_rel_, (**C**) TLR, and (**D**) ∆TLR_rel_. ∆SUV_rel_, relative change in SUV_max_; ∆TLR_rel_, relative change in TLR

As reflected by SUV_max_, collectively, [^89^Zr]Zr-PSMA-617 uptake (Fig. 4A) and changes in that variable over time (Fig. 4B) distinctly differed between the 6 previously-indeterminate lesions that were classified as positive on [^89^Zr]Zr-PSMA-617 PET/CT versus their 14 counterparts that were classified as negative. In the positive lesions, radiotracer uptake rose markedly from the 1-hr to the 24-hr scan, and then essentially plateaued at a high level through 48 h post-injection. In the negative lesions, the already very low degree of [^89^Zr]Zr-PSMA-617 uptake at 1

hr post-injection declined in the subsequent two measurements, or the lesions did not show any clear radiotracer uptake. Additionally, TLR, a marker of lesional contrast, was markedly higher in the [^89^Zr]Zr-PSMA-617-positive lesions than in the [^89^Zr]Zr-PSMA-617-negative lesions at 24 and 48 h (Fig. 4C). Furthermore, in distinction with findings in the negative lesions, TLR increased continuously over time in the positive lesions (Fig. 4D).

**Fig. 5:**
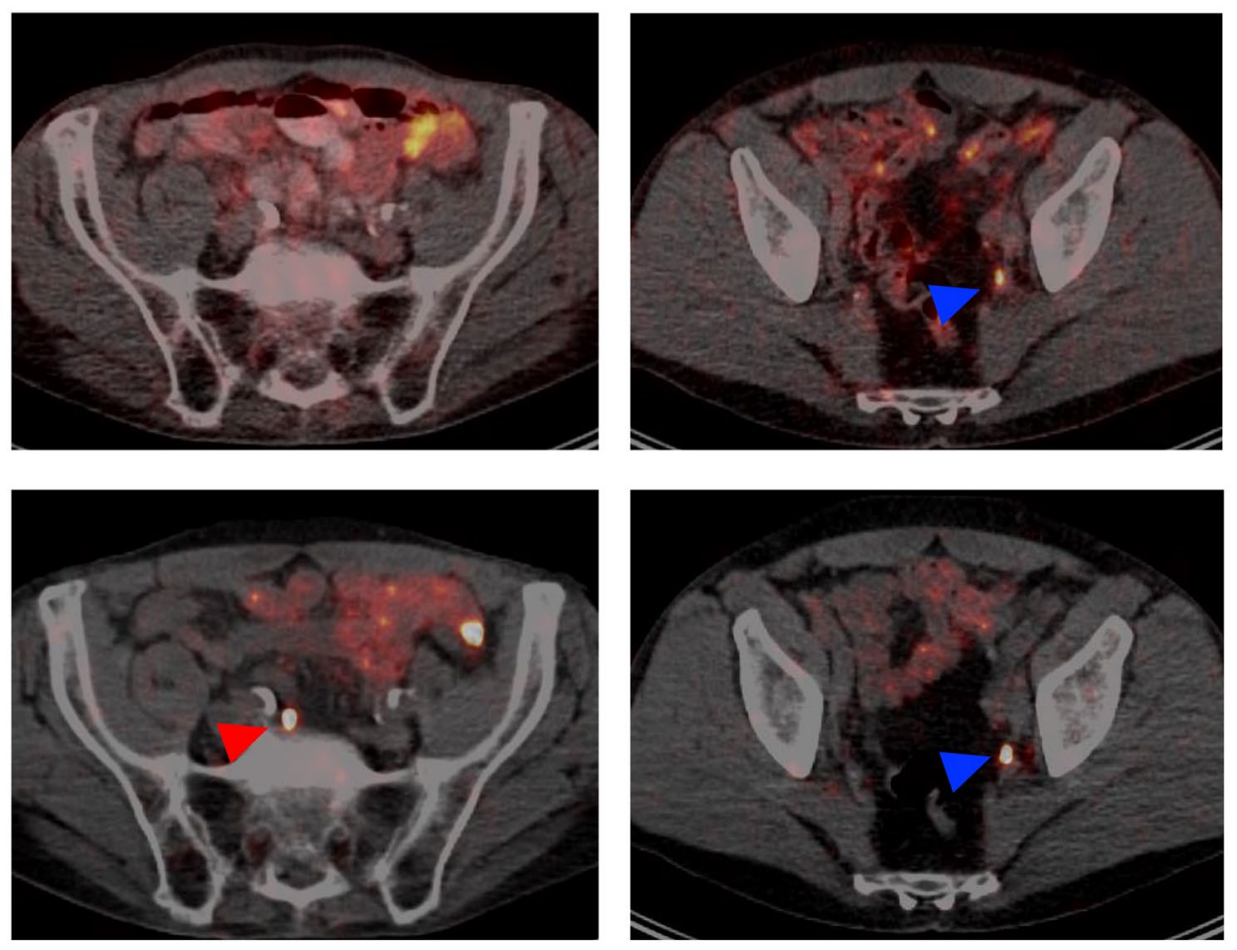
Representative transversal slice images, from two patients (one per column). The left-hand column shows a lesion (red arrow, bottom image) suspicious for lymph node metastasis of prostate cancer that was detected by [^89^Zr]Zr-PSMA-617 PET/CT (48-hr scan shown here) but not [^68^Ga]Ga-PSMA-11 PET/CT (top image). As exemplified in the right-hand column, all lesions detected on [^68^Ga]Ga-PSMA-11 PET/CT also were detected on [^89^Zr]Zr-PSMA-617 PET/CT (lesions indicated by blue arrows)

**Fig. 6:**
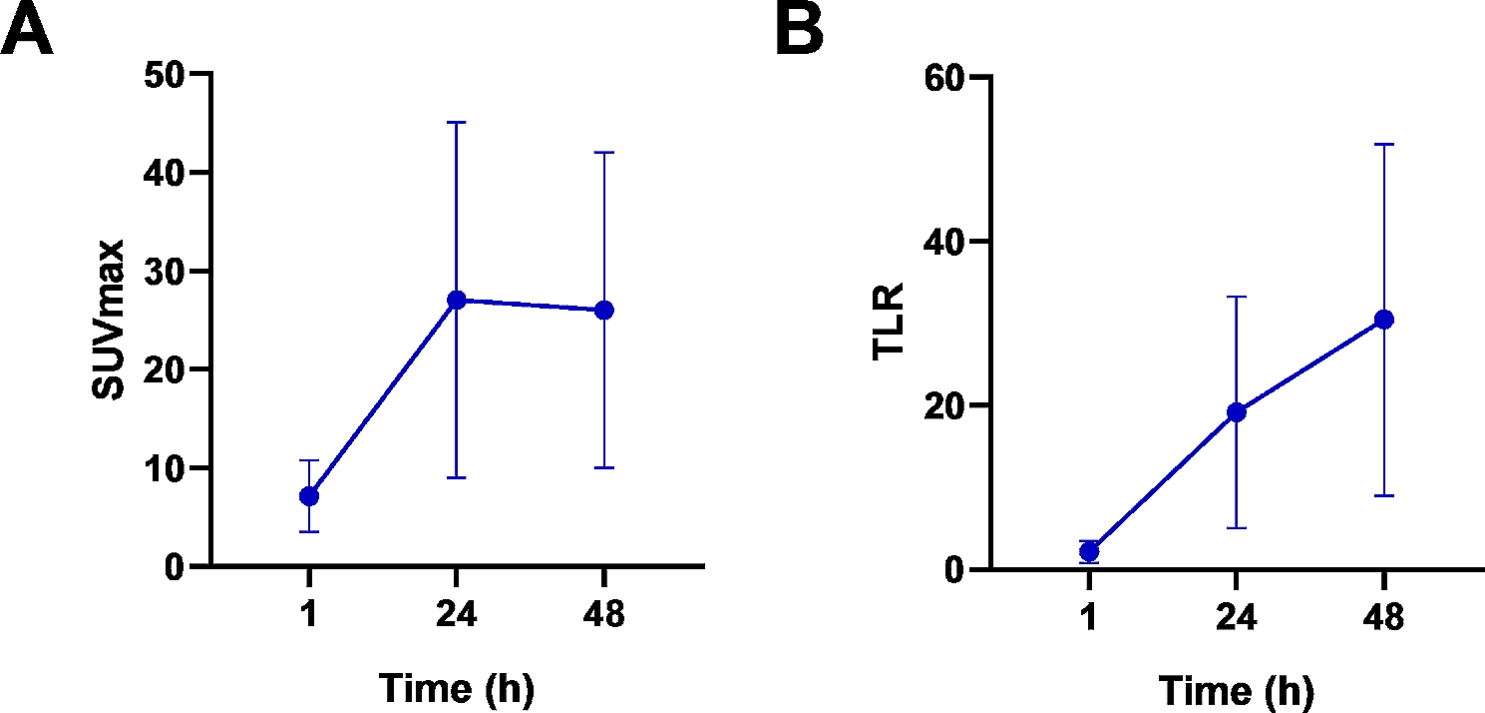
[^89^Zr]Zr-PSMA-617 PET variables by scan time of lesions, which were already suspicious on the [^68^Ga]Ga-PSMA-11 PET/CT: (**A**) SUV_max_ and (**B**) TLR

[^89^Zr]Zr-PSMA-617 PET/CT also identified altogether 11 lesions suspicious for prostate cancer that had not been visualized at all on [^68^Ga]Ga-PSMA-11 PET/CT (Table 2; representative images in Fig. 5, left column). Of the newly-discovered suspicious lesions, 3 were presumed to be local recurrences, and 8, lymph node metastases. Altogether 7/15 patients (47%) had lesions newly found on [^89^Zr]Zr-PSMA-617 PET/CT. Every lesion that was positive on [^68^Ga]Ga-PSMA-11 PET/CT also was clearly seen on the [^89^Zr]Zr-PSMA-617 scans (representative images in Fig. 5, right column). Figure 6 shows the SUVmax and TLR of the [^89^Zr]Zr-PSMA-617 PET/CT of these lesions, which were already suspicious on the [^68^Ga]Ga-PSMA-11 PET/CT. The kinetics of these parameters were similar to those of the [^68^Ga]Ga-PSMA-11 indeterminate lesions, which were classified as positive by [^89^Zr]Zr-PSMA-617 PET/CT.

During the [^89^Zr]Zr-PSMA-617 PET/CT procedure and the 4 wks thereafter, no adverse events, including clinically-relevant vital signs abnormalities, that appeared to be related to the imaging procedure were noted.

Subsequently to [^89^Zr]Zr-PSMA-617 PET/CT imaging, 12/15 (80%) patients received a [^89^Zr]Zr-PSMA-617 PET/CT-guided radiotherapy, 1/15 (7%) androgen deprivation therapy (ADT) and 1/15 (7%) PSMA-targeted radioligand therapy. The remaining one decided to wait and postpone treatment. After [^89^Zr]Zr-PSMA-617 PET/CT-guided radiotherapy PSA serum level decreased by 84 ± 26%; in 6/12 (50%) patients PSA levels were below the detection limit.

## Discussion

This is, to our knowledge, the largest analysis (*N* = 15) published to date assessing the ability of [^89^Zr]Zr-PSMA-617 PET/CT to characterize as suspicious or non-suspicious for prostate cancer lesions that were indeterminate on recent prior [^68^Ga]Ga-PSMA-11 PET/CT. The analysis had three main findings. First, even in the setting of BCR with low PSA levels, and across the three major types of putative prostate cancer recurrence, i.e., prostate bed, lymph node, and bone lesions, previously-indeterminate foci appeared to be readily amenable to such dichotomization on 24-hr and 48-hr [^89^Zr]Zr-PSMA-617 PET/CT images. This observation suggests that, using this novel method might in many cases solve an important diagnostic dilemma associated with conventional PSMA-targeted imaging. Our observations in additional patients align with our preliminary experience in 3 men with indeterminate conventional PSMA-targeted imaging findings [13].

Second, negative and positive lesions showed distinctly different patterns of [^89^Zr]Zr-PSMA-617 kinetics. This observation was reflected by lesional radiotracer uptake, represented by SUV_max_, and by lesional contrast, represented by TLR, as well as by patterns of change in these variables over the 1 to 48 h post-injection. The PET kinetics of the [^68^Ga]Ga-PSMA-11 indeterminate lesions, which were classified as positive by [^89^Zr]Zr-PSMA-617 PET/CT were similar to those of the clearly suspicious lesions on [^68^Ga]Ga-PSMA-11, strengthening the assumption of correct classification by [^89^Zr]Zr-PSMA-617 PET/CT.

Lastly, [^89^Zr]Zr-PSMA-617 PET/CT that was performed to characterize previously-indeterminate lesions not infrequently had incidental but clinically-relevant findings of additional lesions that had been entirely missed on [^68^Ga]Ga-PSMA-11 PET/CT. This observation further supports the efficacy of PSMA-targeted PET/CT with ^89^Zr tracers in localizing sources of BCR, that has been documented in all preliminary analyses published to date [11–16].

Classification of indeterminate conventional PSMA-targeted imaging findings is highly clinically relevant, as this additional information can significantly influence treatment decisions. These decisions may involve the selection of a therapeutic modality or modalities, as well as the regimen of the treatment(s) chosen. Our results suggest that depending on the [^89^Zr]Zr-PSMA-617 PET/CT findings, a precise, individually-adjusted, targeted intervention may be feasible, i.e., metastasis-directed therapy [18–21]. In our cohort, 12/15 patients could receive such a targeted radiotherapy based on [^89^Zr]Zr-PSMA-617 PET/CT with adequate biochemical response. On the other hand, our confirmation of some indeterminate findings as non-suspicious suggests that [^89^Zr]Zr-PSMA-617 PET/CT also might help spare certain patients from over-treatment, including, for example, unnecessarily large radiation fields. As seen here and in our earlier reports [11, 12, 14], [^89^Zr]Zr-PSMA-617 PET/CT appears to be safe. Moreover, the potential benefit of the additional information provided by the procedure would seem to clearly outweigh the main apparent downside of this PET imaging modality, its radiation exposure, which, at ∼ 10 mSv [13], is ∼ 2–3 times higher than that of PET with [^68^Ga]Ga-PSMA-11 or other short-lived PSMA-targeted PET tracers [8, 22–24].

Limitations of this work should be noted. First, the analysis was retrospective and observational, and involved a small, single-center series, weakening strength of evidence and decreasing generalizability. Further studies in larger cohorts, ideally with a randomized, prospective design, are recommended.

Second, in the day-to-day practice setting reported here, no lesion had its malignancy evaluated histopathologically and no follow-up imaging was available. Also, because in this context, experimental imaging could not be applied before conventional imaging, [^68^Ga]Ga-PSMA-11 in all cases preceded [^89^Zr]Zr-PSMA-617 PET/CT. Additionally, more information (results of follow-up during the interval between scans and [^68^Ga]Ga-PSMA-11) was available when interpreting the [^89^Zr]Zr-PSMA-617 images versus the conventional scans. Further, due to the sometimes weeks-long interval between these scans, it cannot be excluded that disease progression may partly accounted for the increased clarity of the previously-indeterminate lesions and/or for the additional lesions seen on the [^89^Zr]Zr-PSMA-617 images. Moreover, the kinetics of benign lesions on [^68^Ga]Ga-PSMA-11 should also be analyzed on [^89^Zr]Zr-PSMA-617 PET/CT in future studies.

Furthermore, it should be noted that we did not evaluate safety of [^89^Zr]Zr-PSMA-617 PET/CT over the long term. Reassurance is provided, though, by the lack of long-term side effects noted to date [11–14], and by the favorable safety profile of zirconium-labeled radiopharmaceuticals deployed in other settings [25–28].

Despite these limitations of our data, and although this analysis must be considered hypothesis-generating, our results suggest that [^89^Zr]Zr-PSMA-617 PET/CT may prove to be a beneficial imaging intervention that can be offered to patients with BCR not only as a complementary procedure in cases of negative conventional PSMA-targeted PET/CT, but also to better characterize indeterminate findings of conventional scans.

## Conclusions

[^89^Zr]Zr-PSMA-617 PET/CT appears to allow characterization of lesions that were previously indeterminate on [^68^Ga]Ga-PSMA-11 PET/CT as suspicious or non-suspicious for prostate cancer. [^89^Zr]Zr-PSMA-617 radiotracer kinetics differ markedly between previously-indeterminate lesions classified into these categories. Because of this ability to differentiate, the potential to identify lesions that entirely elude detection using conventional PSMA-targeted imaging, and the apparent safety of this novel procedure, [^89^Zr]Zr-PSMA-617 PET/CT appears to be a promising imaging method.

## Data Availability

The datasets analyzed during the current study are available from the corresponding author on reasonable request.

## Abbreviations

∆SUV_rel_: Relative change in maximum standardized uptake value
∆TLR_rel_: Relative change in tumor-to-liver ratio
^18^F: Fluorine-18
^68^Ga: Gallium-68
^89^Zr: Zirconium-89
ADT: Androgen deprivation therapy or antiandrogen therapy
CT: Computed tomography
max.: Maximum
min.: Minimum
MIP: Maximum intensity projection
p.i.: Post-injection
PET: Positron emission tomography
PSA: Prostate-specific antigen
PSMA: Prostate-specific membrane antigen
SD: Standard deviation
SUV_max_: Maximum standardized uptake value
SUV_mean_: Mean standardized uptake value
TLR: Tumor-to-liver ratio of SUV_max_: SUV_max_ of presumed tumor lesion/SUV_mean_ of healthy liver
